# Emergence of multi-acaricide resistant *Rhipicephalus* ticks and its implication on chemical tick control in Uganda

**DOI:** 10.1186/s13071-015-1278-3

**Published:** 2016-01-04

**Authors:** Patrick Vudriko, James Okwee-Acai, Dickson Stuart Tayebwa, Joseph Byaruhanga, Steven Kakooza, Edward Wampande, Robert Omara, Jeanne Bukeka Muhindo, Robert Tweyongyere, David Okello Owiny, Takeshi Hatta, Naotoshi Tsuji, Rika Umemiya-Shirafuji, Xuenan Xuan, Masaharu Kanameda, Kozo Fujisaki, Hiroshi Suzuki

**Affiliations:** Department of Veterinary Pharmacy, Clinics and Comparative Medicine, School of Veterinary Medicine and Animal Resources, College of Veterinary Medicine, Animal Resources and Biosecurity, Makerere University, P. O Box 7062, Kampala, Uganda; Central Diagnostic Laboratory, College of Veterinary Medicine, Animal Resources and Biosecurity, Makerere University, P. O Box 7062, Kampala, Uganda; National Research Center for Protozoan Diseases, Obihiro University of Agriculture and Veterinary Medicine, Inada-Cho, Obihiro, Hokkaido 080-8555 Japan; Drug Information Department, National Drug Authority, P. O. Box 23096, Kampala, Uganda; Directorate of Research and Graduate Training, Makerere University, P.O Box 7062, Kampala, Uganda; Laboratory of Parasitic Diseases, National Institute of Animal Health, National Agricultural and Food Research Organization, 3-1-5 Kannondai, Tsukuba, Ibaraki 305-0856 Japan; Present Address: Department of Parasitology, Kitasato University School of Medicine, Kitasato, Minami-ku, Sagamihara, Kanagawa 252-0374 Japan; Present Address: Japan International Cooperation Agency, Nibancho Center Building, 5-25, Nibancho, Chiyoda-ku, Tokyo, 108-8012 Japan

**Keywords:** Ticks, *Rhipicephalus appendiculatus*, *Rhipicephalus* (*Boophilus*) *decoloratus*, Acaricide, Resistance, Amitraz, Synthetic pyrethroids, Organophosphates

## Abstract

**Background:**

Acaricide failure has been on the rise in the western and central cattle corridor of Uganda. In this study, we identified the tick species associated with acaricide failure and determined their susceptibility to various acaricide molecules used for tick control in Uganda.

**Methods:**

In this cross sectional study, tick samples were collected and identified to species level from 54 purposively selected farms (from 17 districts) that mostly had a history of acaricide failure. Larval packet test was used to screen 31 tick populations from 30 farms for susceptibility at discriminating dose (DD) and 2 × DD of five panels of commercial acaricide molecules belonging to the following classes; amidine, synthetic pyrethroid (SP), organophosphate (OP) and OP-SP co-formulations (COF). Resistance was assessed based on World Health Organization criteria for screening insecticide resistance.

**Results:**

Of the 1357 ticks identified, *Rhipicephalus* (*Rhipicephalus*) *appendiculatus* and *Rhipicephalus* (*Boophilus*) *decoloratus* were the major (95.6 %) tick species in farms sampled. Resistance against SP was detected in 90.0 % (27/30) of the tick populations tested. Worryingly, 60.0 % (18/30) and 63.0 % (19/30) of the above ticks were super resistant (0 % mortality) against 2 × DD cypermethrin and deltamethrin, respectively. Resistance was also detected against COF (43.3 %), OP chlorfenvinphos (13.3 %) and amitraz (12.9 %). In two years, 74.1 % (20/27) of the farms had used two to three acaricide molecules, and 55.6 % (15/27) rotated the molecules wrongly. Multi-acaricide resistance (at least 2 molecules) was detected in 55.2 % (16/29) of the resistant *Rhipicephalus* ticks and significantly associated with *R. decoloratus* (*p* = 0.0133), use of both SP and COF in the last 2 years (p < 0.001) and Kiruhura district (*p* = 0.0339). Despite emergence of amitraz resistance in the greater Bushenyi area, it was the most efficacious molecule against SP and COF resistant ticks.

**Conclusion:**

This study is the first to report emergence of super SP resistant and multi-acaricide resistant *Rhipicephalus* ticks in Uganda. Amitraz was the best acaricide against SP and COF resistant ticks. However, in the absence of technical interventions, farmer-led solutions aimed at troubleshooting for efficacy of multitude of acaricides at their disposal are expected to potentially cause negative collateral effects on future chemical tick control options, animal welfare and public health.

**Electronic supplementary material:**

The online version of this article (doi:10.1186/s13071-015-1278-3) contains supplementary material, which is available to authorized users.

## Background

Ticks are one of the leading vectors of diseases of economic importance to the livestock industry in Africa [1–3]. Tick-borne diseases (TBD) mainly theileriosis/East Coast fever (ECF), babesiosis and anaplasmosis present serious constraints to productivity of especially exotic cattle and their crosses. In Uganda, over 30 % of calf crop is lost to TBD [4]. The above diseases also account for nearly 90 % of total disease control costs and over 60 % of total farm inputs [5]. To address tick challenge, commercial cattle farmers rely extensively on acaricides for chemical control of ticks. This has created a huge demand and market for acaricides in Uganda. The liberalization of the veterinary drug industry in the country has made acaricides even more accessible to farmers [6]. Because of limited control, cases of irrational use of acaricides by farmers have been widely reported [6–8]. Wrong dilution, application methods and increased acaricide pressure are amongst factors that accelerate development of acaricide resistance [9, 10]. Acaricide resistance was first reported in Uganda in 1970 against organochlorine toxaphene by *Rhipicephalus* (*Boophilus*) *decoloratus* and *Rhipicephalus evertsi* [11]. The lack of tick acaricide resistance monitoring system since early 1990’s to date implies that the performance of various molecules on the Ugandan market are unknown. However, the increased cases of farmers’ complaints on acaricide failure, especially in western and central cattle corridors, raises serious suspicion of possible emergence of acaricide resistant ticks in the country. In the rest of the world, tick resistance to various classes of acaricides has been extensively reported [10]. Most of the studies reported acaricide resistance against one class of molecules. However, cases of multiple acaricide resistance by *Rhipicephalus (Boophilus) microplus* have been reported in Mexico [12]. Several methods have been proposed for detection of acaricide resistance. Larval packet test (LPT), larval tarsal test (LTT) and adult immersion test (AIT) are among the common tests used [13–16]. However, the limitations associated with the above tests such as high labour and time requirements have resulted in the introduction of genomic based approaches [16–19]. Nevertheless, the high costs of genomic approaches leaves LPT as the most used tool for routine acaricide resistance screening. This is further consolidated by the greater agreement between LPT and genomic tools [16, 20]. The current study established the common species of ticks associated with acaricide failure, acaricide use practices and determined the acaricide resistance profile of the ticks using LPT.

## Methods

### Study area

The primary study area for this research were cattle farms in western and central Uganda that were experiencing acaricide failure between December 2013 and January 2015. Western and central Uganda have the highest population of exotic cattle (especially dairy breeds) and their crosses [21]. Due to the susceptibility of the improved breeds, farmers have to rely on extensive use of acaricides for tick control and prevention of TBD. A total of 14 districts from central and western Uganda were included in this study. They were identified during an earlier investigation of complaints of acaricide failure by the National Drug Authority of Uganda and our research team. Thus, the farms from central (16 farms) and western (34 farms) Uganda were purposively sampled based on history of acaricide failure reported to the respective district veterinary office and animal health workers. However, 4 additional samples were obtained from 1 district in the north (Gulu) and 2 districts in the eastern (Serere and Mbale) parts of Uganda. The sample from Gulu was collected from cattle in the abattoir to establish possible spread of resistant ticks through cattle trade. The tick samples from Mbale were collected electively for purposes of finding a reference susceptible tick. Overall ticks were collected from 54 study sites designated as farms in this study (Additional file 1: Figure S1).

### Tick collection

Ticks were collected from 6 to 20 randomly sampled cattle per farm although dogs were also included for tick collection in farms that had dogs. Ticks were collected from goats and sheep on one farm in Kampala. Cattle, goats and sheep were restrained and inspected for ticks in the various predilection sites. Dogs on the other hand were restrained by the owner before tick samples were picked. Both engorged and semi-engorged ticks from each farm were carefully picked and put in perforated labelled sample bottles and transported to the Central Diagnostic laboratory (CDL) at the College of Veterinary Medicine, Animal Resources and Biosecurity (COVAB), Makerere University for taxonomic identification, hatching and acaricide efficacy assays.

### Taxonomic identification of tick samples

Ticks were identified to species level based on morphological features described by Walker *et al.* [22]. For each farm, identified ticks were categorized based on their species to determine the dominant species associated with acaricide failure at farm and district levels. The engorged female ticks were immediately transferred into individual tubes and incubated at 27 ± 1 °C and 80 % relative humidity for oviposition. After hatching, the larvae were kept in the incubator until they were 18 days old and used for acaricide efficacy assays.

### Acaricides used for tick resistance assay

Commercial acaricide formulations that represented all the classes of acaricide on Ugandan market were purchased from the local importers and used for LPT. They were coded as; A4 (12.5 % amitraz, Kenya), SP3 (10 %, α-cypermethrin, Kenya), SP10 (5 %, deltamethrin, Tunisia), OP (100 %, chlorfenvinphos, Italy), COF1 (co-formulation, 30 % chlorfenvinphos and 3 % α-cypermethrin, Italy). The commercial (brand) names of the acaricides used were coded for anonymity to avoid any misinterpretation as promotion or demotion of such products based on their efficacy result.

### Tick bioassays for acaricide efficacy

A total of 31 tick populations from 30 farms were tested for acaricide susceptibility. For logistical reasons, we adopted the method proposed for insecticide resistance testing by World Health Organization (WHO) [23]. The manufacturers recommended concentration was considered as the diagnostic/discriminating dose (DD) for all the chemicals. However, one additional dose level, which was twice the above dose (2 × DD) was also applied. The diluent used for all the acaricides was trichloroethylene and olive oil mixed in a ratio of 2:1 [24]. For amitraz, the method by Miller *et al.* [25] was used. Briefly, 0.25 mg/ml (DD) and 0.5 mg/ml (2 × DD) commercial amitraz were prepared using the diluent. For cypermethrin and deltamethrin, 0.05 mg/ml (DD) and 0.1 mg/ml (2 × DD) respectively were prepared. For OP-chlorfenvinphos 0.5 mg/ml (DD) and 1 mg/ml (2 × DD) were prepared for the bioassays. The concentration of the coformulation prepared were 0.3:0.03 mg/ml (DD) and 0.6:0.06 mg/ml (2 × DD).

The choice of substrate used for impregnation of the chemicals was based on Food and Agriculture Organization (FAO) [24] recommendation. Filter paper (Whatman No.1, Whatman, Madstone, United Kingdom) was used as a substrate for cypermethrin, deltamethrin, chlorfenvinphos and co-formulated acaricide. Nylon fabric was used for amitraz. The substrates were labelled with pencil and impregnated with 0.7 ml of the corresponding acaricide solution prepared. Trichloroethylene was evaporated in a fume hood for 2 hours. Each impregnated filter paper or nylon fabric was folded into a packet and loaded with, on average, 60 larvae from the same farm and same species. The packets were then secured with alligator clips and incubated at 29 ±1 °C and 80 % relative humidity for 24 hours. Each experiment was carried out in duplicate. In all the assays, contamination was avoided by starting every experiment with the negative control followed by the lower concentration and changing gloves between different acaricide molecules. In the absence of laboratory reference susceptible *Rhipicephalus* ticks in the country, *Haemaphysalis leachi* and *Amblyoma variegatum* larvae that were 100 % susceptible to all the acaricides were taken as reference ticks for phenotypic acaricide resistance assay. The reliability of this approach was later verified using 6 populations of susceptible *R. appendiculatus* and *R. decoloratus* reference ticks collected from low acaricide pressure farms in Adjumani district-north western Uganda (Unpublished data).

After 24 hours, the packets were removed in the order in which they were loaded in the incubator. Three independent enumerators who were previously trained on identifying dead and live ticks using a magnifying lens and stereo-microscope counted the number of ticks that died and those that were alive for each set of experiments. Mortalities were expressed as percentage of the total number of larvae exposed to the acaricide. There were no mortalities recorded in the control groups that were exposed to only the diluent.

### Data on acaricide application practices

A semi-structured interview with farmers and/or farm workers was carried out from 52 of the 54 farms since data could not be retrieved from the two farms. The data captured included breeds of cattle reared, sequence of acaricide brands used in the last two years, method of acaricide application, dilution of acaricide(s) used, application interval at the time of the study and mixing of two or more acaricide formulations at one time. The data on sequence and brands of acaricides were used to determine the correctness of rotation from one molecule to another. Rotation was considered wrong if a farmer changed acaricide brand within the same molecule and changing from COF to SP following acaricide failure. However, a change from synthetic pyrethroid (SP) to co-formulation (COF) and organophosphate (OP) molecule following acaricide failure to SP was also considered a wrong rotation due to the possible cross-resistance between SP and OP [10, 26, 27]. The farm data on acaricide usage was also used to establish the brand preference for the different acaricide molecules on the market. The registration status of various brands of acaricides stated by the farmers was either established from National Drug Authority or verified using the National Drug Authority’s Veterinary Register (http://www.nda.or.ug/docs/Vet_List.pdf).

### Data analysis

The mortality data for the 31 tick populations tested were recorded in MS excel and mean mortality and standard error determined. The WHO [23] percentage mortality cut-off values for susceptibility and resistance against insecticides determined using DD were used to categorize the mortality data. Ticks that showed at least 80 % mortality were considered susceptible while those that showed less than 80 % mortality against a given chemical were considered resistant. The above data together with the qualitative data on acaricide use was analysed using SPSS version 21 (IBM SPSS Statistics for Windows, Version 21.0. Armonk, NY: IBM Corp.). Pearson chi square analysis was done with MedCalc for Windows, version 12.5 (MedCalc Software, Ostend, Belgium) to determine the factors associated with multiple acaricide resistance at 95 % confidence and *p* value ≤0.05 was considered statistically significant.

### Ethical considerations

The study was approved by the institutional review board (No. VAB/REC/15/104) of the College of Veterinary Medicine, Animal Resources and Biosecurity, Makerere University. To ensure biosecurity of ticks, all experiments were carried out under strict in-house procedure for avoiding escape of larvae. All materials used were either autoclaved or soaked in hot water at 99 °C. Larvae that were kept for further molecular studies were preserved in 70 % ethanol. The commercial (brand) names of all the acaricides were coded to ensure confidentiality.

## Results

### Farm characteristics and tick species identified

Of the 54 cattle farms from which ticks were collected, 83.3 % (45/54) kept crosses of exotic cattle and 9/54 had only local cattle as the main livestock enterprise. Up to 90.4 % (47/52) of the farms used hand spray for acaricide application while only 3.8 % (2/52) used plunge dip and another 1.9 % (1/52) used spray race. Complaint of acaricide failure was reported in 94.4 % (51/54) of the farms that were all located in central and western Uganda. A total of 1357 ticks were identified from the 54 study farms. *Rhipicephalus* ticks accounted for 95.6 % (1297/1357) of the tick populations although *A.variegatum* and *H. leachi* constituted 3.5 % (48/1357) and 0.9 % (12/1357), respectively. Amongst the *Rhipicephalus,* 55.1 % (715/1297) were the one host ticks *R. decoloratus* compared to 44.9 % (582/1297) three host tick *Rhipicephalus appendiculatus.* On the other hand, 70.8 % (34/48) of the *A. variegatum* ticks were from eastern Uganda (Table 1). Only one out of the 12 *H. leachi* was collected on cattle, the rest were from dogs. No *Rhipicephalus* tick was found on dogs. For the 51 farms that had complaints of acaricide failure, 98.0 % (1257/1283) of the ticks belonged to the genus *Rhipicephalus. Rhipicephalus (Boophilus) decolortus* were 55.7 % (714/1257) and 42.3 % (543/1257) were *R. appendiculatus. A. variegatum* formed only 1.1 % (14/1257) of the ticks from the 51 farms.

**Table 1:** Species of ticks identified from the various study areas

			Number and frequency (%) per district								
			*R. appendiculatus*		*R. decoloratus*		*A. variegatum*		*H. leachi*		
Region	District	No.farms	No.	%	No.	%	No.	%	No.	%	Total
Central	Kampala	1	17	100.0	0	0.0	0	0.0	0	0.0	17
	Kiboga	1	6	60.0	4	40.0	0	0.0	0	0.0	10
	Kyankwanzi	1	1	9.1	4	36.4	2	18.2	4	36.4	11
	Mpigi	1	30	100.0	0	0.0	0	0.0	0	0.0	30
	Mubende	1	0	0.0	35	100.0	0	0.0	0	0.0	35
	Nakasongola	1	15	48.4	9	29.0	7	22.6	0	0.0	31
	Sembabule	7	79	73.1	29	26.9	0	0.0	0	0.0	108
	Wakiso	3	31	52.5	28	47.5	0	0.0	0	0.0	59
	Mbale	2	1	2.9	1	2.9	33	94.3	0	0.0	35
East	Serere	1	0	0.0	13	92.9	1	7.1	0	0.0	14
North	Gulu	1	38	97.4	0	0.0	1	2.6	0	0.0	39
West	Bushenyi	4	3	0.9	347	99.1	0	0.0	0	0.0	350
	Kiruhura	12	28	17.8	121	77.1	0	0.0	8	5.1	157
	Mbarara	6	96	61.5	56	35.9	4	2.6	0	0.0	156
	Mitoma	3	20	29.4	48	70.6	0	0.0	0	0.0	68
	Rukungiri	8	217	95.6	10	4.4	0	0.0	0	0.0	227
	Sheema	1	0	0.0	10	100.0	0	0.0	0	0.0	10
Total	17	54	582	42.9	715	52.7	48	3.5	12	0.9	1357

### Acaricide molecules and brand preferences by farmers

The veterinary drug register showed that a total of 25 commercial brands of acaricides had been marketed in Uganda. Synthetic pyrethroids (SP1-SP15) constituted 60.0 % (15/25) of the total commercial brands marketed, followed by amitraz (A1-A7 brands) 28.0 % (7/25), co-formulation (COF1-COF2) 8.0 % (2/25) and only one brand of mono-formulated organophosphate was registered. However, 68.0 % (17/25) of the commercial brands of acaricide registered were found to have been used in the study farms. Overall, amitraz accounted for 36.9 % (48/130) of the total acaricide formulations used for tick control followed by COF 30.0 % (39/130), SP 27.7 % (36/130) and mono-formulated OP 5.4 % (7/130) being the least used class of acaricide. Within the same molecule, clear brand preferences were recorded. For example two brands of amitraz (A3 and A4), four brands of SP (SP1, SP2, SP3 and SP13) and 1 brand of COF (COF1) were preferred by 75.0 % (36/48), 69.4 % (25/36) and 71.8 % (28/39) of the farmers, respectively. The majority of the farmers (81.3 %, *n* = 48) used at least two classes of acaricides within the last 2 years. Acaricide registration pattern showed that the rapid influx of different acaricide brands began in 1997 and its climax was attained in 2007. Between 1997 and 1998, all the three classes of acaricides (amidine, SP and OP) were on the Ugandan market suggesting that they have been in use for over 16 years in Uganda (Table 2).

**Table 2:** Acaricide molecules registered in Uganda and report of their use by the farmers

	Brand names, total number and % freq.	Generic name	Dilution (acaricide (ml: water (liters))	Concentration (%)	Freq. of use by farmers in study area	% within class	Overall %	Year licensed by NDA
Classification								
Amidine	A1	Amitraz	2:1	12.5	1	2.1	0.8	2000
	A2	Amitraz	2:1	12.5	8	16.7	6.2	2001
	A3	Amitraz	2:1	12.5	25	52.1	19.2	1998
	A4	Amitraz	2:1	12.5	11	22.9	8.5	1997
	A5	Amitraz	2:1	12.5	3	6.3	2.3	1997
	A6	Amitraz	2:1	12.5	0	0.0	0.0	1998
	A7	Amitraz	2:1	12.5	0	0.0	0.0	2007
Sub-total	7 (28.0)				48	100.0	36.9	
Synthetic Pyrethroid	SP1	α-Cypermethrin	1:1	5.0	8	22.2	6.2	2002
	SP2	α-Cypermethrin	1:1	5.0	5	13.9	3.8	1998
	SP3	α-Cypermethrin	1:2	10.0	7	19.4	5.4	2009
	SP4	α-Cypermethrin	1:1	7.0	3	8.3	2.3	2011
	SP5	Cypermethrin	1:1	10.0	1	2.8	0.8	1998
	SP6	Cypermethrin	1:1	10.0	0	0.0	0.0	1998
	SP7	Cypermethrin	1:1	15.0	0	0.0	0.0	2007
	SP8	Cypermethrin	1:1	10.0	0	0.0	0.0	2005
	SP9^a^	Deltamethrin	1:1	5.0	2	5.6	1.5	-
	SP10	Deltamethrin	1:1	5.0	4	11.1	3.1	2007
	SP11	Deltamethrin	1:1	5.0	0	0.0	0.0	-
	SP12^b^	Flumethrin	-	-	0	0.0	0.0	1997
	SP13	Flumethrin	1:1	2.0	5	13.9	3.8	1997
	SP14	Flumethrin	1:1	2.0	0	0.0	0.0	2010
	SP15	Cyhalothrin	1:1	5.0	1	2.8	0.8	2013
Sub-total	15 (60.0)				36	100.0	27.7	
Organophosphate	OP (1(4))	Chlorfenvinphos	1:2	100	7	100.0	5.4	1997
Co-formulation	COF1	Chlorfenvinphos + α-cypermethrin	1:2	30:3	28	71.8	21.5	2004
	COF2	Chlorpyriphos + Cypermethrin	1:2	50:5	11	28.2	8.5	2013
Sub-total	2 (8.0)				39	100.0	30.0	
Total	25 (100)				130		100	

^a^deregistered, ^b^pour-on, (-) No information’

### Strength variation of SP acaricides sold on the Ugandan market

As shown in Table 2, all (100 %) of the amitraz brands available on the market had a concentration of 12.5 % (wt/vol.). However, 13/15 of the brands of synthetic pyrethroids licensed as emulsified concentrates had concentrations ranging from 2 to 15 %. The 38.5 % (5/13) of synthetic pyrethroid brands were 5 % (wt/vol.) followed by 10 % wt.vol (4/13), 2 % wt/vol. (2/13), 7 % wt/vol (1/13) and 15 % wt/vol (1/15). Moreover, aside from one molecule, the rest were prescribed in a dilution ratio of acaricide to water of 1 ml: 1liter, giving a wide concentration range for chemical tick control in Uganda. Similarly, the two co-formulations on the market had a wide concentration range despite the same dilution ratio of acaricide to water of 1:2 (Table 2).

### Susceptibility of tick larvae against the various molecules used

The percentage mortality of larvae against the different acaricides used in the bioassay at DD and 2 × DD is shown in Table 3. Based on the WHO criteria, 93.5 % (29/31) of the tick populations tested had resistance to at least one class of acaricide molecule. Acaricide resistance was detected in *Rhipicephalus* ticks only.

**Table 3:** Percentage mortality of larvae against various classes of acaricides determined with LPT

District	Farm/Pop. ID	Tick species	% Mortality (Mean ± SEM)									
			Amitraz (mg/ml)		Cypermethrin (mg/ml)		Deltamethrin (mg/ml)		Chlorfenvinphos (mg/ml)		Chlorfenvinphos/cypermethrin (COF) (mg/ml)	
			0.25	0.5	0.05	0.1	0.05	0.1	0.5	1.0	0.3/0.03	0.6/0.06
Kampala	C1	*R. app.*	100 ± 0.0	100 ± 0.0	0	0	0	0	100 ± 0.0	100 ± 0.0	100 ± 0.0	100 ± 0.0
Wakiso	C2	*R. app.*	100 ± 0.0	100 ± 0.0	0	0	0	0	100 ± 0.0	100 ± 0.0	14.7 ± 0.4	100 ± 0.0
	C3	*B. decol.*	100 ± 0.0	100 ± 0.0	0	0	0	0	67.5 ± 0.5	94.5 ± 0.5	21.0 ± 5.0	39.0 ± 3.0
Mubende	C4	*B. decol.*	100 ± 0.0	100 ± 0.0	0	0	0	0	100 ± 0.0	100 ± 0.0	70.4 ± 0.7	93.7 ± 0.8
Mpigi	C5	*R. app.*	100 ± 0.0	100 ± 0.0	11.0 ± 0.0	11.5 ± 0.5	0	12.5 ± 2.5	82.5 ± 5.5	100 ± 0.0	79.0 ± 1	87.5 ± 2.5
Kiboga	C6	*R. app.*	100 ± 0.0	100 ± 0.0	0	0	0	0	100 ± 0.0	100 ± 0.0	98.85 ± 1.2	100 ± 0.0
Gulu	N1	*R. app.*	100 ± 0.0	100 ± 0.0	0	0	0	0	100 ± 0.0	100 ± 0.0	100 ± 0.0	100 ± 0.0
	N2	*A. vari.*	100 ± 0.0	100 ± 0.0	100 ± 0.0	100 ± 0.0	100 ± 0.0	100 ± 0.0	100 ± 0.0	100 ± 0.0	100 ± 0.0	100 ± 0.0
Mbarara	W1	*R. app.*	100 ± 0.0	100 ± 0.0	0	0	0	0	100 ± 0.0	100 ± 0.0	100 ± 0.0	100 ± 0.0
	W2	*B. decol.*	100 ± 0.0	100 ± 0.0	0	0	0	0	50.6 ± 2.7	92.5 ± 0.6	0	19.6 ± 0.4
	W3	*R. app.*	100 ± 0.0	100 ± 0.0	0	0	0	0	100 ± 0.0	100 ± 0.0	71.0 ± 0.0	92.0 ± 0.5
	W4	*R. app.*	100 ± 0.0	100 ± 0.0	6.8 ± 1.1	36.6 ± 7	0	46.5 ± 11.5	100 ± 0.0	100 ± 0.0	93.95 ± 0.9	100 ± 0.0
Kiruhura	W5	*B. decol.*	100 ± 0.0	100 ± 0.0	0	0	0	0	68.7 ± 3.8	76.50 ± 9.8	25.05 ± 0.6	57.1 ± 11.3
	W6	*B. decol.*	100 ± 0.0	100 ± 0.0	0	0	0	19.7 ± 10.3	74.45 ± 3.9	91.2 ± 4.0	62.7 ± 5.7	93.3 ± 2.9
	W7	*H. leach.*	100 ± 0.0	100 ± 0.0	100 ± 0.0	100 ± 0.0	100 ± 0.0	100 ± 0.0	100 ± 0.0	100 ± 0.0	100 ± 0.0	100 ± 0.0
	W8	*B. decol.*	100 ± 0.0	100 ± 0.0	0	0	0	0	100 ± 0.0	100 ± 0.0	66.5 ± 2.2	76.3 ± 0.7
	W9	*B. decol.*	100 ± 0.0	100 ± 0.0	0	0	0	0	100 ± 0.0	100 ± 0.0	56.0 ± 1	65 ± 2.5
Bushenyi	W10	*B. decol.*	68.1 ± 1.9	74.5 ± 1.5	0	10.8 ± 1.5	0	16.3 ± 2.5	92 ± 0.5	96.7 ± 0.9	96.2 ± 1.6	98.8 ± 0
	W11	*B. decol.*	100 ± 0	100 ± 0.0	0	0	0	0	80.2 ± 0.15	100 ± 0.0	49.3 ± 2.7	60.0 ± 1.6
Mitoma	W12	*B. decol.*	100 ± 0	100 ± 0	5.0 ± 0.0	13.5 ± 1.5	8.0 ± 3.0	15.5 ± .5	100 ± 0	100 ± 0	53.5 ± 0.5	100 ± 0
	W13	*B. decol.*	41.5 ± 0.5	62.5 ± 1.5	65.5 ± 1.5	72.0 ± 2.0	73.5 ± 1.5	91.5 ± 2.5	100 ± 0.0	100 ± 0.0	100 ± 0.0	100 ± 0.0
	W14	*B. decol.*	45.0 ± 1.0	NT	NT	NT	NT	NT	NT	NT	NT	NT
Sheema	W15	*B. decol.*	100 ± 0.0	100 ± 0.0	0	0	0	0	100 ± 0.0	100 ± 0.0	56.7 ± 0.9	62.7 ± 2.4
Rukungiri	W16	*B. decol.*	100 ± 0.0	100 ± 0.0	0	0	0	0	100 ± 0.0	100 ± 0.0	95.8 ± 0.3	100 ± 0.0
	W17	*R. app.*	100 ± 0.0	100 ± 0.0	0	0	0	0	100 ± 0.0	100 ± 0.0	100 ± 0.0	100 ± 0.0
	W18	*R. app.*	15.4 ± 0.1	16.7 ± 1.3	97.7 ± 0.5	100 ± 0.0	98.3 ± 0.1	100 ± 0.0	100 ± 0.0	100 ± 0.0	100 ± 0.0	100 ± 0.0
	W19	*R. app.*	100 ± 0.0	100 ± 0.0	10.7 ± 0.4	23.2 ± 1.9	12.0 ± 1.3	27.9 ± 0.7	100 ± 0.0	100 ± 0.0	100 ± 0.0	100 ± 0.0
Sembabule	W20	*B. decol.*	100 ± 0.0	100 ± 0.0	15.0 ± 1.0	26.0 ± 3.0	15.5 ± 0.5	24.0 ± 2.0	100 ± 0.0	100 ± 0.0	88.5 ± 0.5	95.0 ± 0.0
	W21	*R. app.*	100 ± 0.0	100 ± 0.0	0	0	0	0	100 ± 0.0	100 ± 0.0	100 ± 0.0	100 ± 0.0
	W22	*R. app.*	100 ± 0.0	100 ± 0.0	0	0	0	0	100 ± 0.0	100 ± 0.0	100 ± 0.0	100 ± 0.0
Serere	E1	*B. decol.*	100 ± 0.0	100 ± 0.0	79.2 ± 4.7	91.7 ± 1.1	78.7 ± 6.1	95.0 ± 1.9	100 ± 0.0	100 ± 0.0	100 ± 0.0	100 ± 0.0

*R. app.* (*Rhipicephalus appendiculatus*); *B. decol.* (*Rhipicephalus (Boophilus) decoloratus*); *A. vari.* (*Amblyoma variegatum)*; *H.leach* (*Haemaphysalis leachi*);COF, coformulation; *NT* not tested due to few larvae, *Pop* Tick population; N1 and N2 are two tick population collected from abattoir (designated as “one farm” for purpose of this study)

#### Resistance to synthetic pyrethroids

**Fig. 1:**
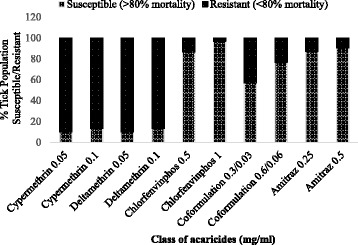
Tick resistance status against various classes of acaricides. Thirty-one tick populations from 31 farms were tested for determining amitraz resistance. Tick resistance to SP, OP and COF were determined using 30 tick populations from 30 farms

At DD, 90.0 % (27/30) of the ticks tested were resistant to both cypermethrin and deltamethrin. Doubling the concentration (2 × DD) of both chemicals did not cause any significant increase in mortality of the above ticks since 86.7 % (26/30) remained resistant (Fig. 1). Moreover at 2 × DD, 60.0 % (18/30) and 63.3 % (19/30) were super resistant (0 % mortality) against cypermethrin and deltamethrin, respectively. Of major concern was the fact that the *R. appendiculatus* collected from cattle in Gulu abattoir (northern region) was among the super resistant ticks (Table 3). Information gathered from the abattoir indicated that cattle from which the *R. appendiculatus* ticks were collected had originated from central Uganda. On the other hand, both *A.variegatum* from Gulu and *H. leachi* from Kiruhura districts were 100 % susceptible at DD for cypermethrin and deltamethrin.

#### Resistance to organophosphate

Mono-formulated OP (chlorfenvinphos) at DD was efficacious in 86.7 % (26/30) of tick populations screened. However, 13.3 % (4/30) of the one host tick *R. decoloratus* were resistant to DD of chlorfenvinphos (Fig. 1). The four tick populations that were resistant were collected from Wakiso, Mbarara and Kiruhura districts.

#### Resistance to co-formulation

At DD of co-formulation, resistance was detected in 43.3 % (13/30) of the tick populations tested. Interestingly, even at 2 × DD, the co-formulated acaricide could not provide the level of effectiveness that was shown by mono-formulated chlorfenvinphos at DD since 23 % (7/30) tick populations tested remained resistant (Fig. 1). Of the 13 *Rhipicephalus* tick populations that were resistant to co-formulation, 76.9 % (10/13) were *R. decoloratus.*

#### Resistance to amitraz

At the DD only 12.9 % (4/31) of the tick populations tested had amitraz resistant *Rhipicephalus* ticks with mortalities ranging from 15.4 to 68.1 %. However, increasing the dose of amitraz to 2 × DD did not result into commensurate level of mortality. Three of the amitraz resistant tick populations were *R. decoloratus* from the greater Bushenyi area (Bushenyi and Mitoma district). One amitraz resistant *R. appendiculatus* tick population was from a farm in Rukungiri district (Table 3). In the current study, amitraz resistance was only recorded in the western part of Uganda.

### Multi-acaricide resistance by *Rhipicephalus* ticks

**Fig. 2:**
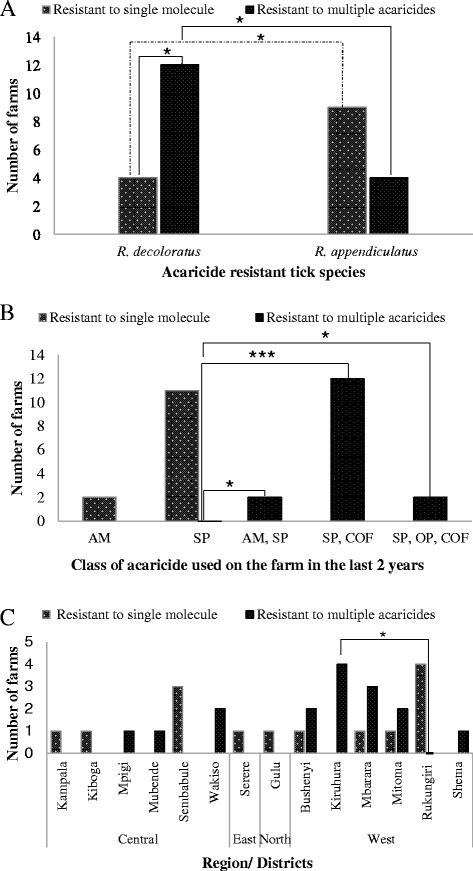
Factors associated with occurrence of multi-acaricide resistance. **a** Tick species associated with multiple acaricide resistance. Comparison of proportion of ticks with single and multiple resistance within each species showed that *R. decoloratus* were significantly associated with multiple acaricide resistance (*p* = 0.0133; 95 % CI = 11.3 % to 75.1 %, *χ* ^2^ = 6.125). Comparison of multiple acaricide resistance between the two tick species showed that *R. decoloratus* was significantly associated with multiple resistance (*p* = 0.0461, 95%CI = 2.9 % to 72.1 %, *χ* ^2^ = 4.020) compared to *R. appendiculatus.* However, *R. appendiculatus* was significantly associated with single resistance when compared to population of *R. decoloratus* resistant to single acaricide molecule (*p* = 0.0461, 95%CI = 2.9 % to 72.1 %, *χ* ^2^ = 3.978). **b** Acaricide molecule resisted by ticks in the farms. Comparison of proportion of farms that used only one molecule (SP) to those that used two to three molecules showed that multiple resistance was associated with use of at least two classes of acaricides; SP, COF (*p* < 0.0001, 95 % CI = 61.1 % to 100 %, *χ* ^2^ = 19.167); AM, SP (*p* = 0.0111, 95 % CI = 11.1 % to 100 %, *χ* ^2^ = 6.453); SP,OP,COF (*p* = 0.0111, 95 % CI = 11.1 % to 100 %, *χ* ^2^ = 6.453). **c** Source (district) of origin of the ticks. Ticks from Kiruhura district were significantly multi-acaricide resistant when compared to those from Rukungiri district (*p* = 0.0339, 95 % CI = 14.8 %–100 %). However, there was no statistical difference in the occurrence of multiple acaricide resistance between the central and western region of Uganda. * = (*p* < 0.05); *** = (*p* < 0.001)

The presence of single or multiple acaricide resistance in the study area is shown in Fig. 2. Resistance to single and multi-acaricide molecules was detected in 48.2 % (13/29) and 55.2 % (16/29) of tick populations from farms with acaricide resistance, respectively. Of the multi-acaricide resistant *Rhipicephalus* ticks, 75 % (12/16) were *R. decoloratus* and the rest were *R. appendiculatus.* Further statistical analysis revealed significant statistical difference (*p* <0.05) in the occurrence of multi-acaricide resistance between the two species of ticks. All the farms that used either SP and co-formulation or SP, OP and COF within the last 2 years had 100 % (14/14) multi-acaricide resistant ticks. There was significant association between use of both SP and COF with resistance to two classes (*p* < 0.001). Kiruhura district had 100 % (4/4) multi-acaricide resistant tick populations, followed by Mbarara (75 %; 3/4) in the western Uganda. Ticks from the two farms in Wakiso district (central Uganda) were also multi-acaricide resistant. Farms that rotated acaricides wrongly had the highest cases of both single and multi-acaricide resistance.

### Farm practices aimed at mitigating acaricide failure

To overcome acaricide failure, various coping strategies have been adopted by farmers although they were considered to potentially worsen the existing tick challenge. Buying different brand(s) of acaricide with little or no regard to similarity in active molecules with previous brand(s) used on the same farm was encountered. In two years, 74.1 % (20/27) of the farms whose tick acaricide resistance status was determined used two to three acaricide molecules, and 55.6 % (15/27) rotated the molecules wrongly. Rotation within the same molecule through purchase of different brands was recorded in 40.7 % (11/27) of the farms. In addition, 25.9 % (7/27) of the farmers increased the concentration of acaricide at least twice over the recommended strength. Some 14.8 % (4/27) of the farmers shortened acaricide application interval to twice a week (every three days). This translates into approximately 10 exposures every month and 120 exposures annually. Mixing of two different acaricide formulations was encountered in 7 % (2/27) of farms and one of the farms mixed co-formulation and amitraz, thus exposing ticks to all the three molecules at once. In a farm that mixed two acaricides and sprayed twice every week, damage to the skin of cattle due to frequent spraying with higher acaricide strength was encountered. As a result, the ticks were easily picked with the damaged skin (Additional file 2: Figure S2).

## Discussion

This is the first report that has comprehensively investigated tick acaricide resistance since the introduction of synthetic pyrethroid, co-formulations and amitraz in Uganda. *Rhipicephalus* ticks are widespread in the country [4], posing a serious threat especially to exotic cattle. Thus TBD especially ECF is ranked by farmers as the most important constraint to cattle production in Uganda [28, 29]. Acaricides are therefore perceived as the most efficient way of controlling ticks and preventing the above diseases. However, with over 25 brands of all the major classes of acaricides circulating on the market (Table 2), farmers are “spoiled for choice”. SP and amitraz accounted for 88 % of the total acaricide brands marketed although amitraz was the most preferred by farmers during the study. This finding is consistent with what was previously reported in north eastern Uganda [30]. Of concern was the variation in strength of the different SP whose dilutions are similar, thus giving different concentrations. It may be possible that amongst cypermethrin, variation in strength may reflect the proprietary difference in composition of *cis* and *trans* isomers. However, there is need for regulatory harmonization of strength of SP formulations with similar active ingredients, notwithstanding inappropriate application practices by farmers. A noticeable example of inappropriate acaricide use was wrong rotation of acaricides between molecules and rotation of acaricides within the same molecule under different brand names. It was also widely believed by farmers that acaricide failure could only be caused by “fake” chemicals. This clearly indicates that farmers lacked knowledge on possibility of ticks becoming resistant to chemicals due irrational acaricide use.

In this study, 93.5 % (29/31) of the larval population tested had resistance to at least one class of acaricide molecule; all of them belonging to the genus *Rhipicephalus*. In Uganda, acaricide resistance was first diagnosed in *Rhipicephalus* ticks against organochlorine, toxaphen in 1970s [11]. This occurred mainly due to increased acaricide pressure considering a compulsory tick control committee enforced weekly dipping of cattle across the country. However, subsequent zoning of acaricides and restricting circulation to the district veterinary office were reported as efficient strategies in delaying acaricide resistance. Nevertheless, political strife in early 1970s [6, 31] and further liberalization of the veterinary drug sector [30] ended both zoning and control in supply of acaricides leading to widespread inappropriate acaricide use. Of major concern now is the high level of resistance to SP (90 %) and emergence of super resistant *R. appendiculatus* and *R. decoloratus* ticks in at least 60 % of the tick populations investigated in this study (Table 3). Since their introduction, SP have enjoyed unique preference due to their dual effect against both ticks and flies [30]. However, its irrational use for over 16 years especially by farmers who use the spray method, could have selected for stable resistance. Studies carried out in related tick-*R. microplus* have attributed such level of resistance to multiple mutations in SP target site, voltage sensitive sodium channel domains II and III [18, 32–34] . A similar level of resistance was first observed in insects and attributed to knock down resistance (*kdr*) in the sodium channel [35–39]. It should be noted that the prevalence of SP resistance by *Rhipicephalus* ticks (96.4 %) reported in this study is amongst the highest compared to those previously known in South America [40–42], India [43] and the rest of Africa [44–47]. Possible evidence of cross-resistance between SP and OP was also observed in 30 % of the tick populations from farms that used co-formulated acaricides. Previous studies in cattle tick showed that ticks that were resistant to SP and OP had elevated esterase activity [26, 48]. The apparent lack of synergism between SP and OP observed in this study possibly emanates from the fact that the most dominant co-formulation used in Uganda (COF1) is prescribed at 1.7 times lower concentration than their corresponding mono-formulations. While the pharmacological basis for such formula is justifiable under ideal conditions, its efficacy is bound to be low in a situation where resistance has emerged against one of the chemicals. This eventually could act as a recipe for emergence of resistance against what otherwise would be the effective molecule (OP) in the co-formulation due to sub-optimal exposure dose. This possibly explains the low efficacy recorded against OP in farms with SP resistant ticks that were also previously exposed to co-formulated acaricides. The mono-formulated OP chlorfenvinphos showed promising efficacy, partly because it is not widely used. The low farm use may be attributed to factors such as shorter application interval recommended for its use and low margin of safety compared to other classes of acaricides. However, emergence of resistance against co-formulation containing OP is an early indication that resistance to this group of acaricides is progressively building amidst fear of possible cross-resistance with SP.

Amitraz resistance was the least detected (12.9 %) in the current study thus corroborating with the findings on in its use at farm level. This finding is consistent with previous studies [49, 50]. Although amitraz formulations have been the dominantly mentioned acaricides (36.9 %), their routine use has remained low due to their narrow spectrum of benefit compared to SP, as far as fly repellence is concerned. This explains why some farmers irrationally mixed amitraz and SP formulations. On the other hand, the increase in amitraz use may be an indicator that farmers were getting better tick control results with amitraz following negative experience while using SP and COF. However, the resistance observed against amitraz in 12.9 % of the tick populations may be mediated by mutation in the amitraz target, octopamine receptor [51–53]. Nevertheless, the high level of multi-acaricide resistance (55.2 %) and emergence of isolated amitraz resistance ticks further emphasizes the need for accelerated intervention to combat their spread across the country. The super SP resistant *R. appendiculatus* collected in Gulu abattoir from cattle bought from central Uganda should be an example of how such ticks can be easily spread through cattle trade and/or movement. Therefore, creation of farm awareness, vigilance amongst veterinarians and cattle traders, and promoting use of amidines in farming communities with ticks that are resistant to SP and coformulation could potentially lead to containment of resistant tick populations. However, the use of amitraz should factor into account the balance between need for tick and tsetse fly control, especially in areas that are known to be tsetse infested as previously reported [30]. In the absence of technical intervention, coping strategies employed by farmers experiencing acaricide failure are likely to worsen the existing challenge. This includes exponential rise in irrational admixing of various acaricide formulations into cocktail and short application intervals that will cause collateral damage to cattle (Additional file 2: Figure S2), food safety and public health. Although alternative technologies such as vaccination of cattle with Muguga cocktail ECF vaccine is being promoted and said to be effective against ECF [54], the emergence of acaricide resistant *R. decoloratus* undermines such efforts. Without controlling the above ticks, babesiosis and anaplasmosis will certainly cause economic losses despite immunization against ECF. Therefore, there is need for various actors in the animal industry to jointly identify strategies for mitigation of acaricide resistance in Uganda. This, however, requires close collaboration between the various stakeholders in the acaricide supply chain and research animal health institutions in the country [55].

## Conclusion

This research is the first in Uganda to report emergence of super SP resistant and multi-acaricide resistant *R. appendiculatus* and *R. decoloratus* ticks. Our results further highlight the importance of routine monitoring of tick acaricide resistance for early detection and intervention especially in countries where veterinary drugs/acaricides are liberalized. In absence of technical interventions, farmer-led solutions aimed at troubleshooting for efficacy of the multitude of acaricides at their disposal are expected to potentially cause negative collateral effect on future chemical tick control options, animal welfare and public health. While understanding the molecular basis of such resistance and countrywide epidemiological studies are necessary, a multi-faceted approach directed towards containment and eradication of acaricide resistant ticks is urgently needed in Uganda.

## Additional files

Additional file 1: Figure S1. Map of Uganda showing the various districts from which tick samples were collected. A, Map of Africa showing Uganda. B, Map of Uganda showing the areas from which ticks were collected (depicted by ticks). (PPTX 656 kb) Additional file 2: Figure S2.
*R. decoloratus* picked from cattle with acaricide induced skin damage. A, Hair bundle (h) that detached from the skin of cattle as the tick (t) was picked; B, Damaged cattle skin (s) that was easily detached with the tick (t); C, The piece of damaged skin (s) firmly attached to the mouth part thus altering the gross morphological appearance of the cephalus region of tick (t); farmer considered these “new” species of ticks. (PPTX 406 kb)
