# Supplementary material for: Emergence of multi-acaricide resistant Rhipicephalus ticks and its implication on chemical tick control in Uganda
Source: Parasit Vectors. 2016 Jan 4;9:4. doi: 10.1186/s13071-015-1278-3 (PMC4700616; doi:10.1186/s13071-015-1278-3)
Supplement: Additional file 1: Figure S1. — Map of Uganda showing the various districts from which tick samples were collected. A, Map of Africa showing Uganda. B, Map of Uganda showing the areas from which ticks were collected (depicted by ticks). (PPTX 656 kb) [file 13071_2015_1278_MOESM1_ESM.pptx]

## Slide 1
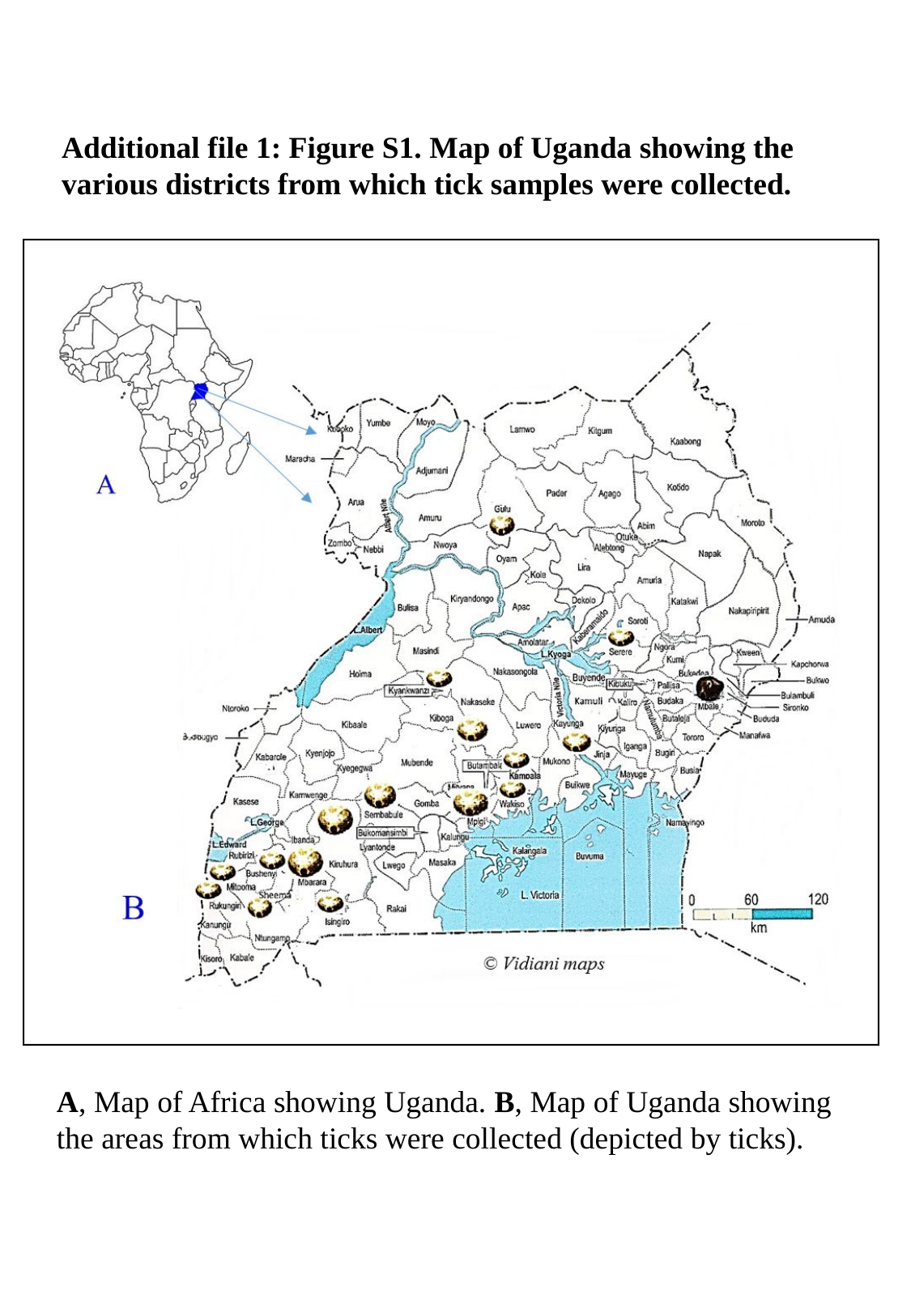

Additional file 1: Figure S1. Map of Uganda showing the various districts from which tick samples were collected.
A, Map of Africa showing Uganda. B, Map of Uganda showing the areas from which ticks were collected (depicted by ticks).
