# Supplementary material for: Emergence of multi-acaricide resistant Rhipicephalus ticks and its implication on chemical tick control in Uganda
Source: Parasit Vectors. 2016 Jan 4;9:4. doi: 10.1186/s13071-015-1278-3 (PMC4700616; doi:10.1186/s13071-015-1278-3)
Supplement: Additional file 2: Figure S2. — R. decoloratus picked from cattle with acaricide induced skin damage. A, Hair bundle (h) that detached from the skin of cattle as the tick (t) was picked; B, Damaged cattle skin (s) that was easily detached with the tick (t); C, The piece of damaged skin (s) firmly attached to the mouth part thus altering the gross morphological appearance of the cephalus region of tick (t); farmer considered these “new” species of ticks. (PPTX 406 kb) [file 13071_2015_1278_MOESM2_ESM.pptx]

## Slide 1
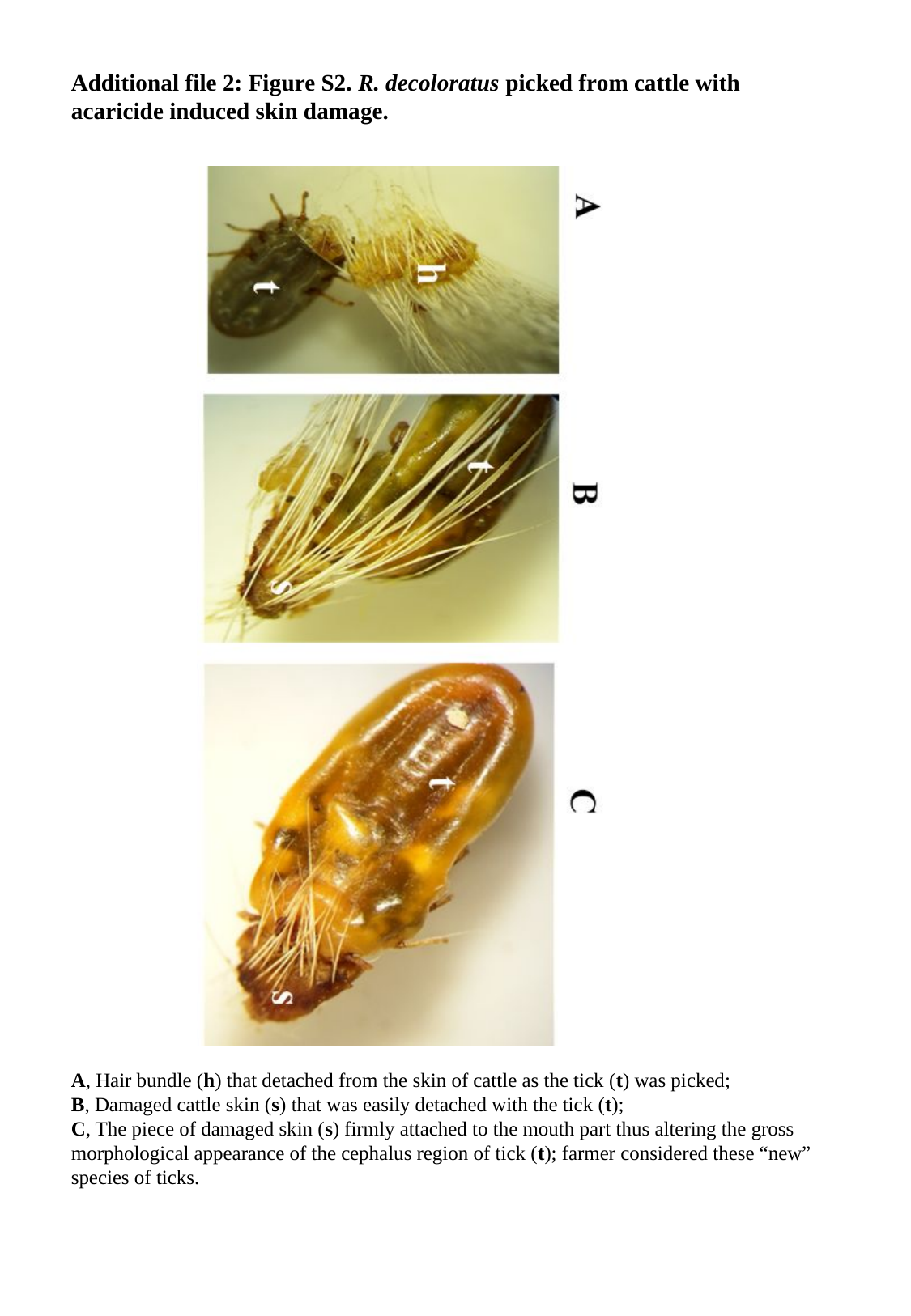

Additional file 2: Figure S2. R. decoloratus picked from cattle with acaricide induced skin damage.
A, Hair bundle (h) that detached from the skin of cattle as the tick (t) was picked;
B, Damaged cattle skin (s) that was easily detached with the tick (t);
C, The piece of damaged skin (s) firmly attached to the mouth part thus altering the gross morphological appearance of the cephalus region of tick (t); farmer considered these “new” species of ticks.
